# Daily Management of Patients on Multikinase Inhibitors’ Treatment

**DOI:** 10.3389/fonc.2022.903532

**Published:** 2022-07-04

**Authors:** Carla Colombo, Simone De Leo, Matteo Trevisan, Noemi Giancola, Anna Scaltrito, Laura Fugazzola

**Affiliations:** ^1^ Department of Endocrine and Metabolic Diseases, IRCCS Istituto Auxologico, Italiano, Milan, Italy; ^2^ Department of Pathophysiology and Transplantation, University of Milan, Milan, Italy

**Keywords:** MKI, thyroid cancer, QoL, Lenvatinib, Vandetanib, Cabozantinib, multidisciplinary tumor board, prehabilitation

## Abstract

In a minority of differentiated thyroid cancer (TC) cases and in a large percentage of poorly differentiated TCs (PDTCs) and anaplastic TCs (ATCs), the prognosis is poor due to the lack of response to conventional treatments. In the last two decades, multikinase inhibitor (MKI) compounds have been developed and demonstrated to be very effective in these aggressive cases. Besides the great efficacy, several adverse events (AEs) have been reported in virtually all patients treated with MKIs, largely overlapping between different compounds and including hypertension, diarrhea, anorexia, decreased weight, fatigue, and proteinuria. Most grade 3–4 adverse reactions occur during the first 6 months of treatment and require dosage reduction and/or drug discontinuation. Due to severity of the AEs related to the treatment with MKIs, a multidisciplinary team is definitely required for the daily management of these patients, for the evaluation of the disease status, and the psychophysical condition. Moreover, it is crucial that the patients could have a facilitated access to reach either specialist doctors or nurses who must have been trained to follow them for their individual clinical complications. The follow-up visits should take place at monthly intervals until the sixth month and then every 1–2 months until the completion of the first year of treatment. The flow chart followed at our tertiary center is reported in the present review as a real-life-based example for the follow-up of patients with advanced TC on MKI treatment.

## Introduction

Thyroid cancer (TC) has an excellent prognosis, since available treatments, namely, surgery and radiometabolic therapy, are very effective. However, in a minority of differentiated TC cases and in a large percentage of poorly differentiated TCs (PDTCs) or anaplastic TCs (ATCs), the prognosis is poor due to the refractoriness to conventional treatments. In the last two decades, multikinase inhibitor (MKI) compounds have been developed and demonstrated to be very effective with a long-lasting response to treatment. Unfortunately, since MKIs are cytostatic, they must be chronically administered to patients in order to avoid a sudden growth of metastases upon discontinuation of treatment (1).

Among the different MKIs approved for TC, Lenvatinib (LEN) and Vandetanib (VAN) are the most widely used for radioiodine-refractory advanced differentiated thyroid cancer (RAI-R DTC) and for medullary thyroid cancer (MTC), respectively.

The registration studies reported excellent results regarding the efficacy of LEN: the SELECT study showed a median progression-free survival (PFS) of 19.4 months in the group of patients treated with LEN compared to 3.7 months in the placebo group (2), being similar and even better results reported in following real-life studies (3–16). Similarly, treatment with VAN demonstrated in the registration ZETA study a great efficacy, showing a median PFS of 30.5 months for the VAN group and a median PFS of 19.3 months in the placebo group (17). Of note, VAN had a higher objective response rate (ORR) with respect to placebo even in patients with sporadic disease and without detectable RET mutations. Subsequently, real-life studies confirmed the efficacy of VAN in terms of both ORR and PFS (18–24). More recently, another MKI, Cabozantinib (CABO), was approved for the treatment of advanced MTC and RAI-R DTC. Data obtained from the registration EXAM study showed that the estimated median disease-free survival (DFS) was approximately threefold higher in the CABO arm than in the placebo group (11.2 vs. 4.0 months, respectively) (25) (**Table 1**).

**Table 1:** The main characteristics of the ZETA, EXAM, and SELECT trials, including eligibility criteria and adverse events (AEs).

Drug	Trial	Trialdesign	Patients(n)	Eligibility criteria	All grades AEs (%)		Grade > 3 AEs (%)	
**Lenvatinib**	SELECT Schlumberger et al., 2015 (2)	Phase III, randomized, double-blind, vs. placebo	261	18 years or older + measurable, pathologically confirmed DTC + 131I-refractory disease	Hypertension Diarrhea Fatigue/asthenia Decreased weight Nausea Stomatitis	67.8 59.4 59 50.2 41 35.6	Fatigue/asthenia Nausea Decreased appetite Decreased weight Hypertension Diarrhea	27.5 13.7 11.5 9.2 9.2 8.4
**Vandetanib**	ZETA Wells et al., 2011 (17)	Phase III, randomized, double-blind, vs placebo	231	Adults + measurable, unresectable, advanced/metastatic MTC + performance status ¾ 2 + serum CT ≥ 500 pg/ml	Diarrhea Rash Nausea Hypertension Headache Fatigue	56 45 33 32 26 24	Diarrhea Hypertension QT prolonged Fatigue Decreased appetite Rash	11 9 8 6 4 4
**Cabozantinib**	EXAM Elisei et al., 2013 (25)	Phase III, randomized, double-blind, vs. placebo	219	Adults + unresectable, advanced/metastatic MTC + disease progression within the prior 14 months	Hypertension Hemorrhage Venous thrombosis GI perforation Non-GI fistula Arterial thrombosis	32.7 25.2 5.6 3.7 3.7 2.3	Hypertension Venous thrombosis Non-GI fistula Hemorrhage GI perforation Arterial thrombosis	8.4 5.6 3.7 3.3 3.3 0.9

Despite the great efficacy, several adverse events (AEs) have been reported in virtually all patients treated with these MKIs and include hypertension, diarrhea, decreased appetite, decreased weight, fatigue, and proteinuria. Most grade 3–4 adverse reactions occur during the first 6 months of treatment and require dosage reduction and/or drug discontinuation (**Table 1**).

Unfortunately, only few data regarding the quality of life (QoL) of patients during MKI therapy have been reported to date. Jasim et al. evaluated QoL of patients treated with LEN by means of the Linear Analog Self-Assessment (LASA) item on a scale of 0–10, and no variations were found at 2 and 6 months of follow-up, but the drug was discontinued in 28% of cases (26). Two Italian studies evaluated the impact of MKIs treatment on daily life and welfare. Nervo et al. used the Patient-Reported Outcomes version of the Common Terminology Criteria for Adverse Events (CTCAE) and the European Quality of Life 5 Dimensions 3 Levels (EQ-5D-3L) questionnaire, providing the EQ-5D index and the EQ visual analogue scale (EQ-VAS). The median EQ-5D index and EQ-VAS scores after 3 months of treatment were lower compared to baseline, but after 12 months, they could be restored, probably as a result of therapy optimization (27). Giani et al. evaluated QoL in 39 patients by using the European Organization for Research and Treatment (EORTC) Quality of Life Questionnaire-Core 30 and the pain visual analogue scale (VAS). Interestingly, no statistically significant differences before, during, and at the end of a 6-month follow-up were found, although a minor improvement of the general health and emotional and cognitive status associated with a slight worsening of the physical role and social functioning was observed (28).

The management of AEs, therefore, emerges as crucial to maintain or improve the eventually decreased QoL of these patients. Few studies evaluated how to optimize the daily management of patients treated with MKIs, suggesting the best approach for clinical practice, aimed to maximally limit dose reductions or treatment discontinuation (29–31).

**Figure 1:**
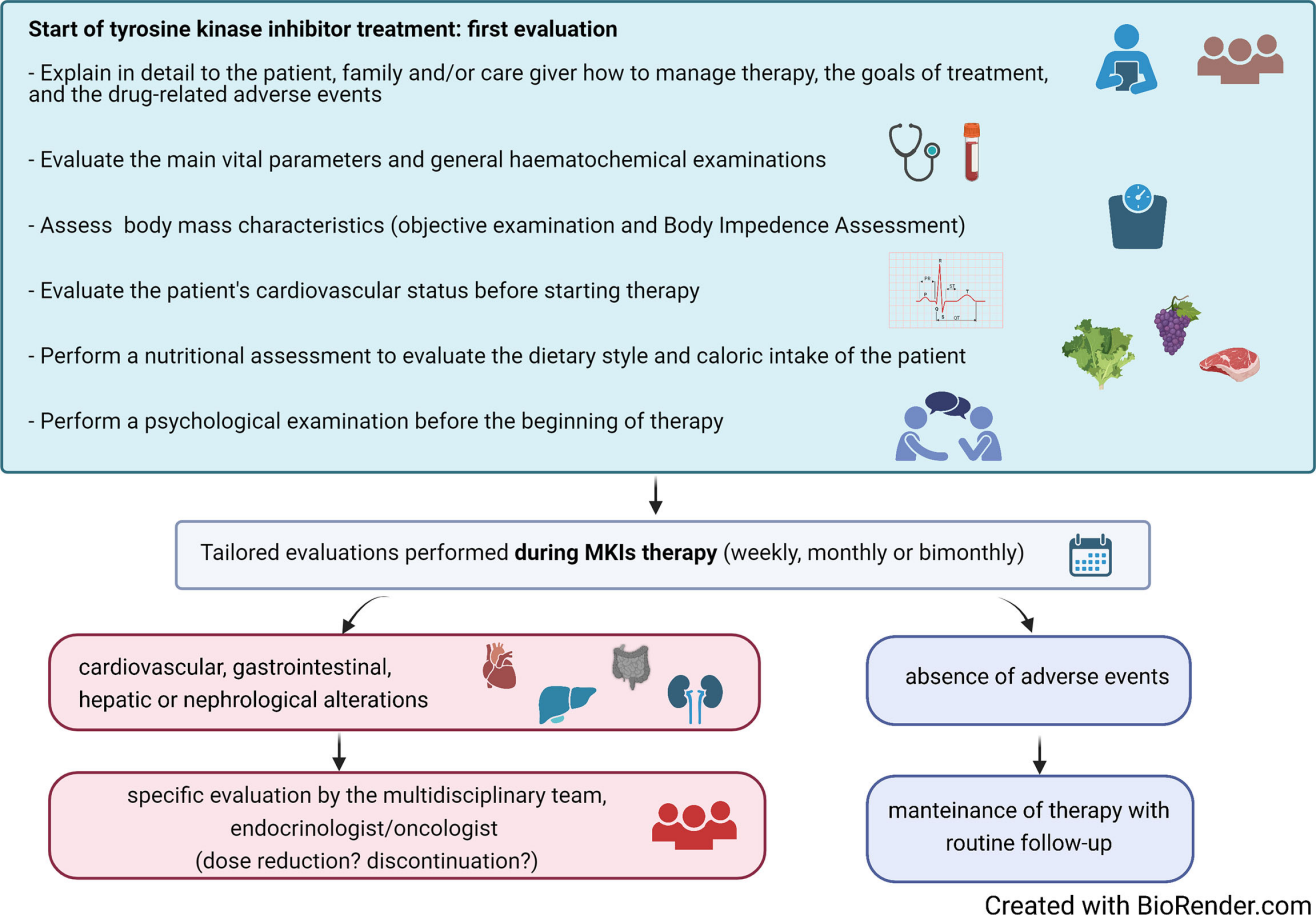
Suggested algorithm for the initial assessment and the follow-up during treatment of patients with advanced thyroid carcinoma treated with multikinase inhibitors (MKIs).

The availability of a multidisciplinary team (MDT) of healthcare professionals, including endocrinologists, oncologists, nurses, physiotherapists, nutritionists, psychologists, and other health professionals, has been shown to facilitate the management of AEs, thus improving the QoL of these fragile patients (32). Upon the identification of a progressive disease that could benefit from a MKI treatment, the patient should receive a careful assessment of her/his psychophysical state. A detailed counseling discussion with the patient and the family should be also planned in order to fully describe risks/benefits of the therapy and the possible occurrence of AEs. This would help the patient to fully share the decision to start a chronic treatment and to identify possible AEs in an early phase (**Figure 1**). Recently, prehabilitation practice has been extended to the management of several tumors, including TC. This includes multidisciplinary interventions that aim to enhance patient’s functional capacity and to reduce side effects of medical or surgical treatments. As far as patients predicted to be submitted to MKIs are concerned, prehabilitation should include the optimization of the nutritional and of the psychophysical status to be obtained in the weeks/months before the start of treatment.

## A Multidisciplinary Team for the Management of the Adverse Events

Several adverse events (AEs) have been reported in almost all patients treated with MKIs, most grade 3–4 adverse reactions occurring during the first 6 months of treatment and requiring dosage reduction and/or drug discontinuation.

Gastrointestinal AEs are frequent and significantly reduce the QoL of patients, also compromising their adherence to treatment. Among these, diarrhea is highly prevalent (range, 45%–67% any grade), and decreased appetite/anorexia, nausea and occasionally vomiting are observed, too. To alleviate these symptoms, clinical practice suggests to train the patients to select among foods and preferentially take those less associated with the onset of gastrointestinal symptoms. Since these aliments can have interindividual major differences and can change during the follow-up, a tailored diet should be prepared and modified according to the inputs given by patients. Natural products or supplements (such as Aloe, lactic ferments, and clay) or loperamide should be suggested in case of diarrhea. Hence, patients need to be followed up at baseline and throughout the treatment by expert nutritionists for the setup of appropriate dietary regimens aimed to reduce or avoid weight loss and gastrointestinal AEs, which can have a major impact on the QoL.

Nevertheless, the majority of patients do develop weight loss during MKI therapy, with a multifactorial basis, including decreased appetite, gastrointestinal disorders, dysgeusia, and modifications of the sense of taste. We recently demonstrated that weight loss is mainly associated with fat mass reduction and that leptin parallels the decrease in body mass index (BMI) values, whereas ghrelin levels increase upon BMI decrease, likely leading to the weight stabilization observed after 1 year of treatment (33). Still, the continuous support of an expert nutritionist is needed, possibly integrated with a fitness rehabilitation program to avoid fat-free mass loss. The latter can be a crucial intervention also for the improvement of fatigue, which is another frequent AE of MKI treatment with a strong impact on the QoL and on the fulfillment of the usual daily activities.

We reported the first demonstration (34), recently confirmed by an independent group (35), that fatigue can be frequently related to primary adrenal insufficiency and that, in patients in whom this diagnosis is confirmed, the use of replacement therapy with cortisone acetate significantly improves the symptom. This intervention increases compliance to therapy, avoiding or limiting dose reductions.

Another important and frequent AE is hypertension, which, in some patients, is easily managed with medical monotherapy, in particular by the use of angiotensin-converting enzyme (ACE) inhibitors (which have a beneficial effect on proteinuria, too), while other cases requires the use of multiple drugs to be controlled (e.g., calcium channel blockers or sartanics or diuretics) (36). These patients should be treated by a cardiologist dedicated to MKI-induced hypertension, who will evaluate them before the start of treatment and during the entire follow-up, with the final aim not to modify antineoplastic treatment due to an unmanageable hypertension.

The majority of MKIs can induce skin alterations of different degrees. It is therefore essential, when the treatment with topical creams (for example those with urea addition to be used for the so-called “hand–foot syndrome”) is not enough to reduce the degree of skin lesions, to submit the patient to a specific dermatological examination. For the treatment of this AE, the support of the nursing staff is crucial. Indeed, nurses are fully involved in the management of these fragile patients, since they usually have a more confidential and tight contact with them, thus providing to the clinician valuable information on the general psychophysical state, the adherence to therapy, and any complaint occurring during treatment.

Finally, a psychological support must be always available in the MDT for all patients, but mostly for those who develop mood variability, anxiety, stress, and depression related not only to the neoplastic condition but also to the coexistence of two or more AEs.

## Daily Management of Patients in MKI Treatment: A Suggested Model

Especially during the first 6 months of MKI therapy, patients should be able to promptly and easily reach either specialist doctors or nurses. Indeed, during the first months, MKIs display their maximum efficacy (37), and acute complications such as fistulization upon tumor necrosis can occur. Moreover, the great majority of AEs develop during the first months of treatment.

**Figure 2:**
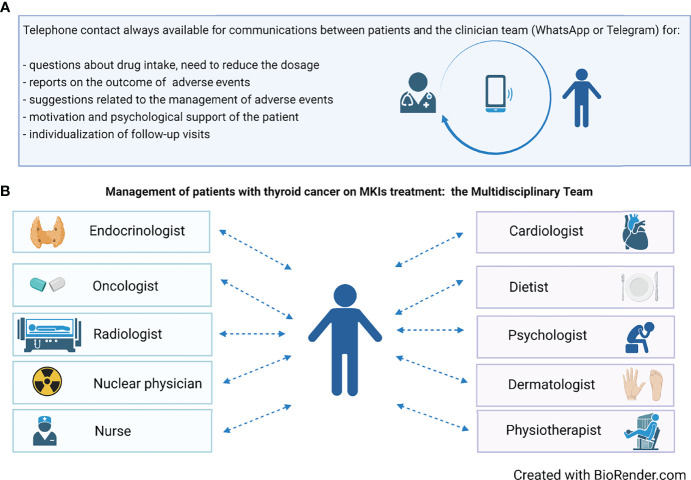
**(A)** The availability of a phone contact is crucial during treatment for an adequate management, aimed to maximally reduce the need for dose reduction or drug discontinuation. **(B)** The composition of the multidisciplinary team involved in the management of patients on MKIs treatment.

To this aim, since the start of MKI therapy, patients should be provided with direct phone and email contacts of the whole medical team. As a consequence, each patient, who is completely evaluated at the hospital every 1–2 months, also has a 24-h direct contact and feels fully supported **(** **Figure 2A** **)**. Even in the absence of complaints, patients like to update the team *via* email, WhatsApp, and Telegram on their health status, and on the positive evolution of AEs. Through these easy contacts, patients can be given dietary advice in case of gastrointestinal AEs, or antihypertensive therapy can be constantly titrated and personalized, thus avoiding a hospital access. In addition, the clinician can monitor on a near-daily basis the body weight’s trend in a patient who has developed decreased appetite or nausea or weight loss on MKI treatment.

The Telegram application in particular allows all physicians in the referral team to simultaneously view patient communications and to quickly provide useful and encouraging feedbacks, with the possibility to act on AEs or any complication in a very early phase. On the other hand, patients feel reassured and more prone not to discontinue treatment even in the presence of AEs. Importantly, this way of communication can be considered a training for the younger members of the team who will learn from the more experts how to daily manage these patients.

Later follow-up can be tailored according to the patient’s general condition. In principle, hospital visits should be scheduled at close intervals during the first treatment period (e.g., every 7–15 days), subsequently reducing frequency to every 30–60 days. At each time of follow-up, AEs not easily manageable by the treating clinicians (endocrinologists/oncologists) will be treated by a specialized physician (cardiologist, dermatologist, etc.) (**Figure 2B**).

## Discussion

MKIs provide a useful therapeutic tool for patients with advanced TC, for whom the prognosis was poor until a few years ago. Despite the great efficacy, major limitations exist, such as the need to maintain a chronic treatment and the need to manage several AEs that highly reduce QoL in some patients. In this context, it is worth to note that studies focusing on patients’ QoL are still very limited and need to be implemented in the next future.

To manage these patients, a multidisciplinary team is crucial. In particular, the decision on the need to start an MKI treatment, when to start it, and at which dosage must be taken mainly by endocrinology/oncology, radiology, and nuclear medicine specialists in a common board. Thereafter, several other specialists should be involved, including psychologists and nurses.

The patient must feel to be followed up and treated by specialists who are expert on this particular topic and must be always supported in the management of AEs in order to increase the compliance and to reduce the risk of discontinuation of the drug.

Upon the identification of a progressive disease that could benefit from a MKI treatment, the patient should receive a careful assessment of her/his psychophysical state and possibly undergo prehabilitation procedures, and a detailed counseling with the family members should be also planned.

During follow-up, it is crucial for the MDT to manage even daily the communication with the patient, mainly thanks to social networks, such as Telegram or WhatsApp, in order to verify the onset and the degree of AEs and to give a prompt advice. These messaging tools allow clinicians to give continuity of care to patients receiving MKIs and to extend their support beyond the periodic visits to the hospital.

In conclusion, the management of MKI treatment must take advantage of an MDT and of the possible daily connection between clinicians and patients. Every effort should be done in this direction to gain the maximum benefit from these very effective drugs and to reduce the AEs, in order to improve compliance to treatment, thus increasing drug effectiveness and patients’ QoL.

## Author Contributions

This mini-review was conceived and written by CC and LF. All authors contributed to the article and approved the submitted version.

## Funding

This study was partially funded by Ricerca Corrente Istituto Auxologico Italiano IRCCS (PTC-array, 05C825_2018 and THY-CANC) and by the Italian Ministry of University and Research (PRIN 2017-2017YTWKWH).

## Conflict of Interest

LF is a consultant for Eisai Europe Limited.

The remaining authors declare that the research was conducted in the absence of any commercial or financial relationships that could be construed as a potential conflict of interest.

## Publisher’s Note

All claims expressed in this article are solely those of the authors and do not necessarily represent those of their affiliated organizations, or those of the publisher, the editors and the reviewers. Any product that may be evaluated in this article, or claim that may be made by its manufacturer, is not guaranteed or endorsed by the publisher.
